# A Systematic Review and Identification of the Challenges of Deep Learning Techniques for Undersampled Magnetic Resonance Image Reconstruction

**DOI:** 10.3390/s24030753

**Published:** 2024-01-24

**Authors:** Md. Biddut Hossain, Rupali Kiran Shinde, Sukhoon Oh, Ki-Chul Kwon, Nam Kim

**Affiliations:** 1 School of Information and Communication Engineering, Chungbuk National University, Cheongju-si 28644, Chungcheongbuk-do, Republic of Korea; hossain.biddut@chungbuk.ac.kr (M.B.H.); rups@chungbuk.ac.kr (R.K.S.); 2 Research Equipment Operation Department, Korea Basic Science Institute, Cheongju-si 28119, Chungcheongbuk-do, Republic of Korea; sukhoonoh@kbsi.re.kr

**Keywords:** deep learning, 3D MRI, transfer learning, federated learning, Swin transformer, MRI datasets, DL tools

## Abstract

Deep learning (DL) in magnetic resonance imaging (MRI) shows excellent performance in image reconstruction from undersampled k-space data. Artifact-free and high-quality MRI reconstruction is essential for ensuring accurate diagnosis, supporting clinical decision-making, enhancing patient safety, facilitating efficient workflows, and contributing to the validity of research studies and clinical trials. Recently, deep learning has demonstrated several advantages over conventional MRI reconstruction methods. Conventional methods rely on manual feature engineering to capture complex patterns and are usually computationally demanding due to their iterative nature. Conversely, DL methods use neural networks with hundreds of thousands of parameters and automatically learn relevant features and representations directly from the data. Nevertheless, there are some limitations to DL-based techniques concerning MRI reconstruction tasks, such as the need for large, labeled datasets, the possibility of overfitting, and the complexity of model training. Researchers are striving to develop DL models that are more efficient, adaptable, and capable of providing valuable information for medical practitioners. We provide a comprehensive overview of the current developments and clinical uses by focusing on state-of-the-art DL architectures and tools used in MRI reconstruction. This study has three objectives. Our main objective is to describe how various DL designs have changed over time and talk about cutting-edge tactics, including their advantages and disadvantages. Hence, data pre- and post-processing approaches are assessed using publicly available MRI datasets and source codes. Secondly, this work aims to provide an extensive overview of the ongoing research on transformers and deep convolutional neural networks for rapid MRI reconstruction. Thirdly, we discuss several network training strategies, like supervised, unsupervised, transfer learning, and federated learning for rapid and efficient MRI reconstruction. Consequently, this article provides significant resources for future improvement of MRI data pre-processing and fast image reconstruction.

## 1. Introduction

Magnetic resonance imaging is an advanced non-invasive medical imaging method with high resolution, which, together with contrast mechanisms, can visualize the anatomy and function of the body [1]. It contributes to medical research and smart healthcare by yielding high-quality reconstructed images without using harmful radiation [2]. However, the image acquisition time [3] of MRI is markedly longer than that of computed tomography. This increases the MRI costs and generates artifacts caused by patient movement. Accelerating MRI acquisition is required to improve patient experiences, enhance clinical workflow efficiency, and enable new imaging capabilities.

Parallel imaging (PI) [4] and compressed sensing (CS) [5] are the two most popular approaches for accelerating MRI acquisition. PI techniques [6,7] offer significant advantages in terms of the scan time reduction and patient comfort while maintaining or improving the image quality. However, they also come with some trade-offs, including the need for calibration data, potential reductions in the signal-to-noise ratio, and sensitivity to various factors that can introduce artifacts. CS-MRI reconstruction works by exploiting the inherent sparsity or compressibility of the underlying image in a certain transform domain. The key idea is to acquire only a subset of k-space data points, typically through significant undersampling, and then reconstruct the full image using a mathematical optimization process. The effectiveness of CS is influenced by the choice of the sparsity transformation domain. The optimal transformation may vary for different types of images and anatomies. In real-time applications, iterative optimization algorithms used in CS reconstruction may face challenges in meeting computational requirements. The combination of CS and PI is a powerful strategy for accelerating MRI scans while preserving the image quality [8,9]. It is particularly valuable in scenarios where significant scan time reductions are required, such as dynamic imaging, functional MRI, or imaging of pediatric or uncooperative patients. However, combining CS and PI may increase sensitivity to certain artifacts, such as residual aliasing artifacts and noise amplification, especially at very high acceleration factors. PI approaches raise the localized noise that has an impact on the reconstruction accuracy and CS depends on the right choice of the regularization penalty and the relevant influences.

Deep learning (DL) has been applied successfully in medical imaging [10,11] such as reconstruction [12], classification [13], segmentation [14], and detection [15]. Conventional feature-extraction approaches require human intervention, and DL directly analyzes the image data. DL-based MRI reconstruction strategies could enhance the flexibility without lessening the image quality. The advantages of deep learning in MRI image reconstruction include the improved reconstruction speed, reduced artifacts, and enhanced image quality, but there are still issues with speed and accuracy. It is also necessary to conduct more research to comprehend the underlying mechanisms of this method. This paper provides a thorough summary of current developments in deep MRI reconstruction to identify these difficulties. In addition, this study examines the field’s opportunities and problems and provides insights into its potential future growth. This review intends to improve knowledge of deep MRI reconstruction and provide an outline for potential studies in this area. However, few studies have reviewed DL-based applications for MRI. Ahishakiye et al. [16] gathered records using DL, image reconstruction, medical imaging, open software, and open imaging data keywords. Montalt-Tordera et al. [17] described existing machine learning (ML) algorithms and their clinical applications. Zhang et al. [18] focused on the mathematical expression of DL algorithms. He et al. [19] analyzed the performance of several contemporary unsupervised learning algorithms, and Knoll et al. [20] reviewed the most significant ML algorithms for parallel imaging based on linear and non-linear approaches. Here, we discuss not only conventional ML-based MRI reconstruction methods but also advanced training strategies—such as the Swin transformer, transfer learning, and federated learning—for rapid and efficient MRI reconstruction.

The objectives of this article are the following:

- Provide an overview of state-of-the-art DL-based MRI reconstruction techniques, including their advantages and disadvantages.
- Describe the potential of transfer learning (TL), and federated learning (FL) approaches for reducing computation complexity and addressing data scarcity and privacy issues in rapid MRI reconstruction.
- Discuss the advantages and challenges of transformer-based (widely used in natural language processing) networks in image capture, information matching, and reconstruction.
- Review the utilities of DL tools, medical-imaging competitions, and open-source codes in MRI.
- Describe publicly available k-space and image datasets for MRI reconstruction and analysis.

The remainder of this article is organized as follows. In Section 2 and Section 3, we explain the survey methodology and several DL frameworks. The DL-based MRI reconstruction methods are reviewed in Section 4. The MRI datasets and open-source codes are described in Section 5. The DL-based MRI reconstruction concerns and future perspectives in this field are set out in Section 6. Finally, the conclusion of this systematic review is described in Section 7.

## 2. Motivation and Methodology

**Figure 1:**
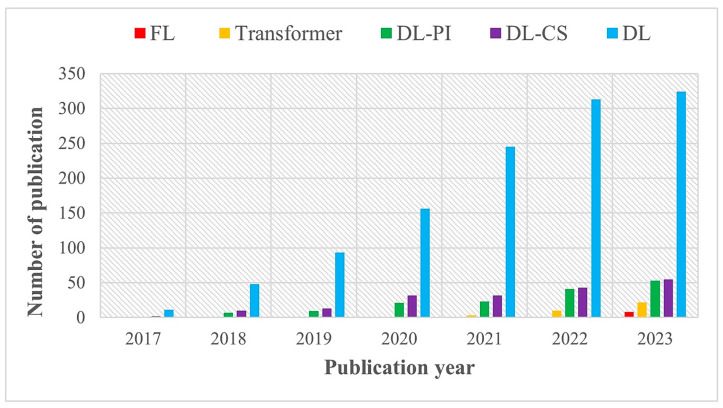
Number of published articles on MRI reconstruction using federated learning (FL), transformer, DL-PI, DL-CS, and DL from PubMed database (2017–2023).

DL networks have successfully recovered MRIs from undersampled measurements by utilizing their capacity to learn efficient models from training data. The trained model is used to reconstruct high-quality images from new, unseen test data. This development generated great attention in relation to DL MRI reconstruction, which prompted continuous improvements in network designs, data augmentation methods, regularization strategies, and loss functions. A fascinating summary of the publishing analysis from January 2017 to November 2023 that focuses on the application of DL models in MRI reconstruction is provided in Figure 1. It reveals the number of annual publications found from the PubMed dataset using five different keywords: deep learning (DL), deep learning-based compressed sensing (DL-CS), deep learning-based parallel imaging (DL-PI), federated learning (FL), and transformer-based MRI reconstruction. These findings show that deep MRI reconstruction performance and generalization are constantly being improved. Deep reconstruction networks, however, are still a developing area of study. Researchers often overlook crucial aspects, like quantitative mapping, super resolution, and magnetic resonance fingerprinting, in favor of focusing largely on DL- and CS-based MRI reconstruction models [21]. This work explores the state-of-the-art of fast and efficient MRI reconstruction using DL algorithms on undersampled k-space datasets.

**Figure 2:**
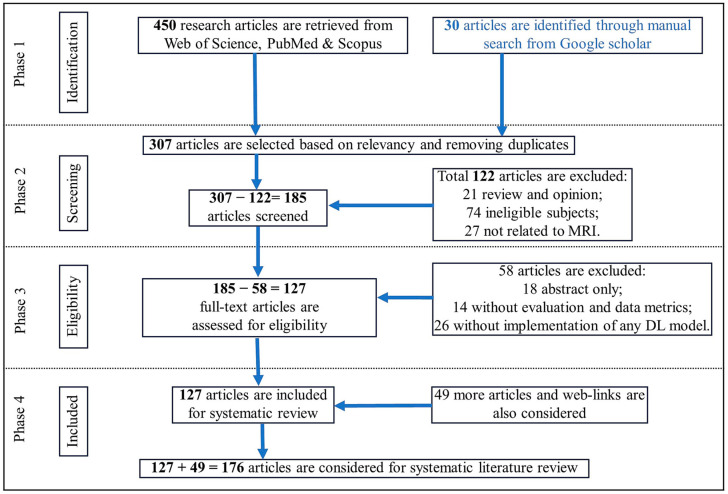
A PRISMA flow diagram illustrating the methodology and criteria for the inclusion and exclusion of research articles.

The preferred reporting items for systematic reviews and meta-analyses (PRISMA) [22] structure and methodology are used to identify the pertinent research articles that are illustrated in Figure 2. The four main phases are: (i) identification: articles collected from various sources; (ii) screening: duplicate and insufficient articles eliminated; (iii) eligibility: analysis of the articles to determine their suitability for review and exclude unsuitable articles; and (iv) inclusion: selection of articles to be included in the study. We examined documents located electronically using four sets of keywords: (i) compressed sensing MRI, deep learning, and magnetic resonance image reconstruction; (ii) federated learning, transfer learning, and magnetic resonance imaging; (iii) Swin transformer, attention mechanism, and medical imaging; and (iv) MRI reconstruction in GitHub, deep learning tools, and MRI data. We performed searches of the Google Scholar, Scopus, Web of Science, PubMed, and MDPI databases and in other journals.

## 3. DL Frameworks and Tools

### 3.1. DL Architectures

Deep neural networks (DNNs) are used for medical-image reconstruction, quality enhancement, feature mapping, contrast transformation, classification of tumors or cancer types, and segmentation for detecting normal and abnormal tissues. Deep architectures can extract features from data in place of conventional hand-crafting feature extraction algorithms. DL can reconstruct high-quality images from undersampled data via discovering complex mappings using undersampled k-space data and fully sampled images. Several DL architectures used for MRI reconstruction are described below.

**Figure 3:**
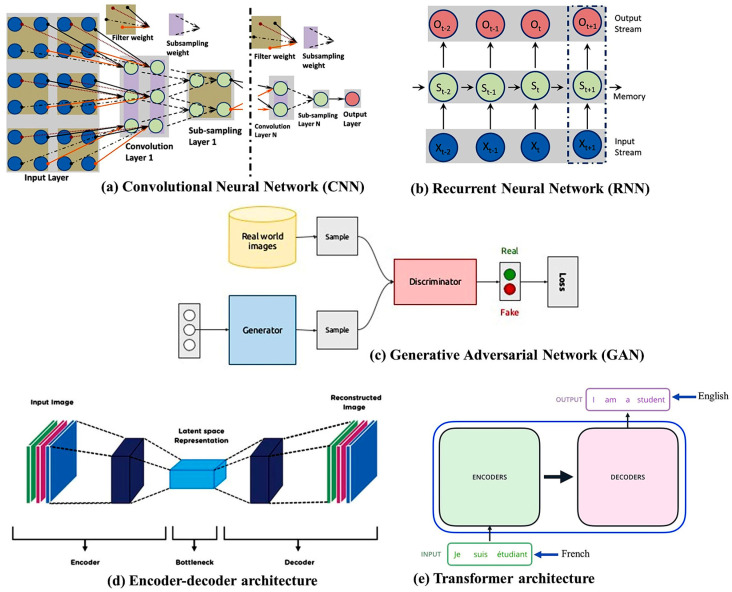
Several deep neural network architectures.

A convolutional neural network (CNN) [23] (Figure 3a) is an efficient approach to DNNs that is particularly effective in image processing and computer vision (CV) tasks. It consists of a set of convolutional layers and applies convolution operations to the input data. These operations involve sliding small filters (kernels) over the input image to learn local features. Through these convolution operations, the network captures low-level features (e.g., edges, textures) in the early layers and progressively more abstract and complex features in the deeper layers. The convolutional layers produce feature maps that represent learned patterns and features in the input data. Thus, CNNs automatically learn hierarchical representations of features in images, making them well-suited for tasks related to images and videos. CNNs have been widely successful in tasks such as image reconstruction, classification, object detection, and segmentation. Google, Microsoft, and Facebook have established research groups to examine novel CNN designs [24]. A CNN deals with raw images and, in some cases, minimizes the data pre-processing tasks. The AlexNet [25], ResNet [26], Squeeze-MNet [27], and Unet [28] networks are typically used in computer vision tasks. However, a CNN needs a large dataset and several layers to understand the global context or relationships between latent features in an image [29].

A recurrent neural network (RNN) [30] (Figure 3b) is a type of artificial neural network (ANN) in which the connections between nodes create a directed graph over time, which is used in sequential data processing. In general, RNNs are applied to sequential data, but they are not the primary choice for sequential image processing. Images are spatial data and the sequential dependencies in pixel values vary across an image. In this case, image data are treated as a time series (e.g., frames of a medical imaging sequence), and RNNs are applied to capture temporal dependencies and variations over time. In MRI reconstruction, RNNs are employed to dynamically adjust the sampling pattern during the acquisition process. However, RNNs are prone to vanishing and exploding gradient problems during training. Long sequences can result in vanishing gradients, where the gradients become very small and hinder learning. Conversely, exploding gradients can cause instability during training. Recently, advanced recurrent architectures, such as long short-term memory (LSTM) and gated recurrent units (GRUs) have been developed to address some of the issues associated with traditional RNNs. Deep RNNs [31] and ConvLSTM [32] models are typically used for image reconstruction and classification.

A generative adversarial network (GAN) [33] (Figure 3c) is more realistic than a CNN and does not require pre-processing. Conversely, this model is more complex than other models, e.g., CNNs and RNNs. A GAN comprises a discriminator and a generator. Given a random variable input, the generator produces data samples. The probability of a particular sample coming from the true dataset is estimated by the discriminator. In the context of MRI reconstruction, GANs can be used to generate realistic and high-quality images from undersampled or noisy MRI data. The generator learns to fill in missing information, generating images that closely resemble the fully sampled counterparts. The discriminator plays a crucial role in distinguishing between generated (reconstructed) images and real images. The discriminator’s objective is to minimize the binary cross-entropy loss function. It learns to assign high probabilities to real images and low probabilities to generated images. The loss is backpropagated through the discriminator to update its parameters. However, training GANs can be unstable, and finding the right balance between the generator and discriminator can be challenging. The training process is sensitive to hyperparameters, and achieving convergence can be difficult. RadialGAN [34] and StarGAN [35] are the most popular GAN architectures.

Encoder–decoder architectures [36] (Figure 3d) are indeed a common and powerful design pattern in various DL applications, including computer vision and natural language processing. These architectures are particularly prevalent in tasks that involve transforming one type of data into another, such as image-to-image translation, sequence-to-sequence tasks, and generative models. The general structure of an encoder–decoder architecture consists of two main components. These encoder–decoder architectures showcase the flexibility and adaptability of the framework for various image reconstruction tasks. Depending on the specific requirements of a task, researchers and practitioners choose or design architectures that best suit the characteristics of the data and the goals of the reconstruction. These architectures are designed to learn the mapping between undersampled or corrupted MRI data and fully sampled or high-quality images. Variations of these architectures [37] are commonly used in the field of medical imaging for tasks like MRI denoising, super-resolution, and artifact correction. However, encoder–decoder architectures may lose fine details during the encoding and decoding process. This can be problematic for tasks that require precise details, such as fine-grained image generation. A variational autoencoder (VAE) [38] is used for MRI reconstruction.

The transformer [39] (Figure 3e) was developed recently and is popular in natural language processing (NLP) based on its even-deeper mapping, sequence-to-sequence model design and adaptive self-attention. Unlike traditional RNN-based models, which process the input sequence sequentially, the transformer is able to process the entire sequence in parallel. The transformer consists of two main modules: the encoder and the decoder. The encoder discovers the input sequence and generates a set of hidden representations, while the decoder uses those representations to generate the output sequence. Both the encoder and the decoder consist of multiple layers of self-attention and feedforward neural networks. One of the key advantages of the transformer is its ability to handle long-range dependencies in the input sequence and its computational efficiency. It has been used for image analysis in terms of object detection [40] and image recognition [41]. The transformer is used in MRI in a variety of ways [42], given its superior capability in image reconstruction and synthesis, as shown in natural images. However, transformers involve a quadratic self-attention mechanism, making them computationally expensive for large inputs. This complexity can be a limitation, particularly when dealing with high-resolution images.

### 3.2. DL Tools

DL tools are used to develop models for generating good results. Several popular open-access DL tools used in MRI processing are listed in Table 1. Among them, TensorFlow and PyTorch are widely used.

### 3.3. Network Training Strategies

#### 3.3.1. Supervised and Unsupervised Learning

Supervised learning is a common technique used in medical image analysis, including the analysis of MRI data. In supervised learning, a machine learning model is trained on a labeled dataset, where each input (in this case, an MRI image) is associated with a corresponding output (typically, a label or annotation). The model learns to map inputs to outputs by identifying patterns and relationships in the training data. Supervised learning in MRI has been applied to a wide range of tasks, including tumor detection and segmentation, disease classification, image registration, and more. It has the potential to significantly enhance the accuracy and efficiency of medical image analysis. However, it also requires large and high-quality labeled datasets and careful validation to ensure its reliability in clinical practice.

Unlike supervised learning, where the algorithm is provided with labeled training data (input–output pairs), unsupervised learning [56] involves working with unlabeled data. The goal of this learning is to find patterns, structures, or representations in the data without specific guidance regarding the output. Unsupervised learning methods [57,58] are particularly valuable when dealing with large and complex MRI datasets, as they can reveal hidden structures and patterns within the data without the need for extensive manual labeling. Real-time 3D MRI reconstruction from cine-MRI using unsupervised networks involves leveraging neural networks to reconstruct dynamic 3D MRI volumes from a sequence of 2D images acquired over time (cine-MRI) [59]. However, the interpretation of the results obtained from unsupervised learning can be more challenging and often requires domain expertise to make meaningful clinical inferences. These methods are an essential part of the toolkit for researchers and clinicians working with MRI data.

Semi-supervised learning [60] is a machine learning paradigm that combines elements of both supervised and unsupervised learning. It is particularly useful when you have access to a small amount of labeled data and a large amount of unlabeled data. It is especially valuable in scenarios where acquiring large amounts of labeled data is challenging. This learning can leverage the available labeled data to improve the model performance on tasks such as classification, segmentation, or regression. Semi-supervised learning in MRI analysis offers the advantage of leveraging both labeled and unlabeled data to enhance model performance. By combining the strengths of supervised and unsupervised learning, semi-supervised approaches have the potential to improve the accuracy and robustness of MRI-based diagnostic and analysis tasks.

Self-supervised learning [61] is an emerging and powerful technique for training machine learning models, especially in scenarios where obtaining labeled data is challenging or expensive. Self-supervised learning is a type of unsupervised learning where the data itself provide supervision for training. This learning in MRI analysis leverages the inherent structure and properties of MRI data to guide the training process, making it a valuable approach for improving the quality of MRI images, enhancing data availability, and addressing various challenges in MRI research and clinical applications. It is an area of active research with the potential to significantly impact the field of medical imaging.

#### 3.3.2. Transfer Learning

**Figure 4:**
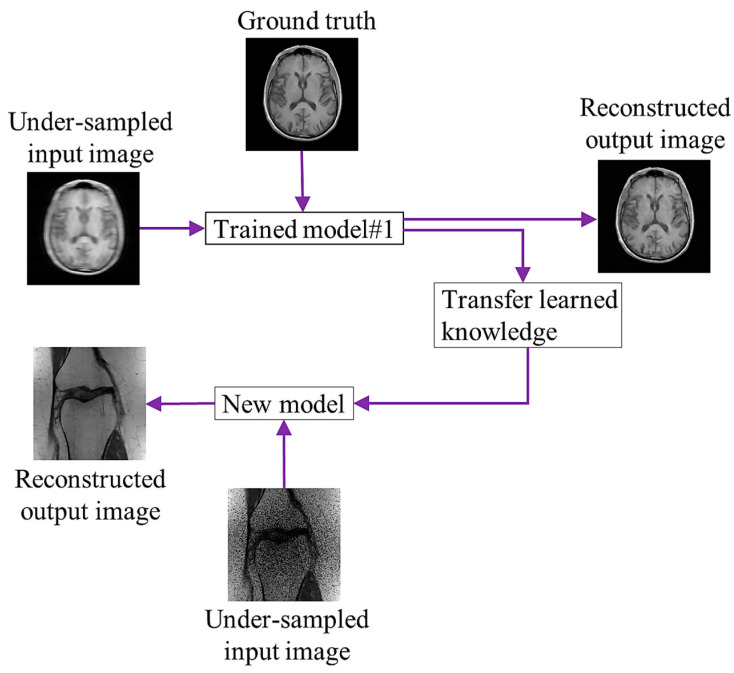
Concept of transfer learning.

Transfer learning (TL) [62] is the process of learning a new activity more effectively by transferring the knowledge acquired in one or more source tasks and applying it to the learning of a related target task. The development of methods for knowledge transfer is a step toward making ML as effective as human learning. Using information from the source task, TL aims to enhance learning in the target task. To improve DL network performance, the model complexity is typically increased by raising the architecture’s numbers of layers and nodes. Multiple model parameters must be accurately learned using a large amount of training data. The performance of a model’s reconstruction is typically improved by adding training data. However, because preserving k-space data is not part of the typical clinical flow, it is challenging to obtain patient raw data for training the network. Consequently, the generalizability of a network based on a few samples needs to be improved. Figure 4 shows a diagram of TL, in which the trained model uses the input and reference brain images for learning. After training, it shares the learning knowledge (weights) with a different model to reconstruct an image of a knee.

A TL strategy addresses the lack of data issues during network training for rapid MRI [63]. For single-channel MRI reconstruction, Arshad et al. [64] assessed a trained Unet on MRIs with different magnetic field strengths, anatomical variations, and undersampling masks. However, none of the studies described above have made use of the generalization ability of multi-channel MRI reconstruction models. The generalizability of a TL-based model for sub-sampled multi-channel MRI reconstruction using GAN has been evaluated [65,66]. Park et al. [67] reported a blended TL technique for both the pre-training and target compressed cardiac cine MRI datasets to mitigate data-privacy concerns. Dynamic dictionaries based on the TL approach [68] employed a limited number of training samples and prior knowledge about the unknown signal to precisely rebuild the image by transferring the existing sample information to the unknown sample. By learning the relationship between the navigator and data slices, Gulamhussene et al. [69] suggested a unique time-resolved four-dimensional (4D) MRI framework based on the same acquisition scheme. In TL, network training is carried out in a domain with many accessible datasets, and information obtained by the trained network is subsequently transferred to a different domain with undersampled data. However, the performance of TL depends on the availability of diverse and representative data during pre-training. If the pre-training data lack diversity in terms of the imaging conditions, patient demographics, or pathology, the transferred knowledge may not effectively address the complexities of the target MRI reconstruction task.

#### 3.3.3. Federated Learning

**Figure 5:**
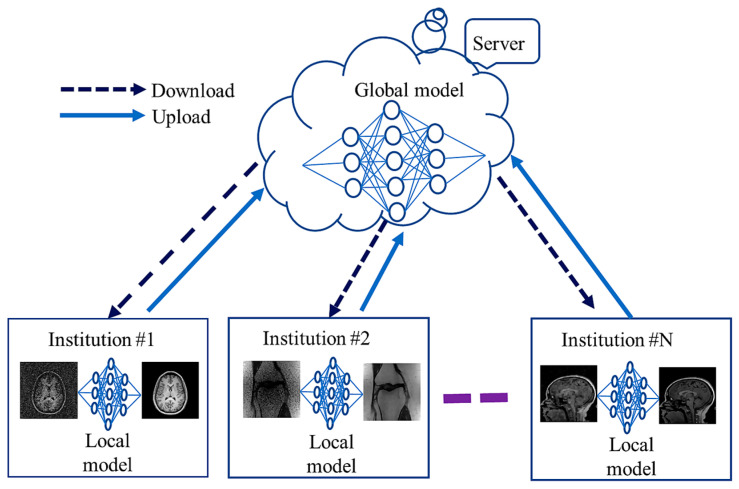
Federated learning for MRI reconstruction.

Deep networks frequently need large amounts of diversely matched data, which can be labor- and cost-intensive to obtain. Furthermore, retaining patients’ data raises privacy concerns, making it challenging to share the information with other institutions. This problem is addressed by the recently developed FL framework [70], which enables the cooperative and distributed training of DL-based techniques. In FL, data are stored locally, and statistical models are trained across segmented data centers or remote devices, e.g., smartphones or hospitals. The training of diverse and possibly large networks poses unexpected problems that call for a fundamental change from conventional methods for large-scale DL, remote optimization, and confidentiality data analysis. To create a global model, a cloud server communicates explicitly with each institution on a regular basis before sharing the data with all the institutions. Each organization uses and maintains its own set of personal information. FL algorithms communicate only about model parameters or update gradients rather than sending actual training data, alleviating privacy concerns. Figure 5 shows communication between global (server side) and local models among several institutions during training. Local models learn from local data and share their weights with the global model.

Li et al. [71] proposed an FL strategy in which shared local model weights are adapted via a randomization procedure while a decentralized iterative optimization process is applied. Their FL framework encompasses two domain algorithms based on the systemic heterogeneity of functional MRI distributions from various sites. Domain shifts between sites in current FL-based MRI reconstruction efforts have not been investigated extensively. To increase the homogeneity of latent-space interpretations in reconstruction approaches, adversarial connectivity between the source and destination sites was suggested by Guo et al. [72]. Feng et al. [73] concentrated on the confidentiality of multi-institutional information in MRI image reconstruction by using the domain shift. Their reconstruction models were divided into a global encoder (used at all sites) and local decoders (individually trained at each site). Elmas et al. [74] suggested a two-stage reconstruction method that involves relating the imaging operator input and cross-site adaptation of a generative MRI baseline. A continuous adversarial model that creates a high-quality image from low-dimensional dependent variables generated by a mapper captures global MRI knowledge. By allowing various institutions to collaborate without having to combine local data, FL can increase data privacy. However, the domain shift of MRI methods can markedly reduce the FL model performance. Levac et al. [75] explored FL for MRI reconstruction by training global models across several clients (data sites) with local scans through employing end-to-end unrolled DL models. An algorithm, FedPR [76], was presented to learn federated visual prompts in the global prompt null space for MRI reconstruction. The review article [77] emphasized the difficulties of using FL in applications related to medical imaging and offered suggestions for future developments. The generalizability of models trained using FL is inadequate [78]; its improvement is a focus of research.

## 4. MRI Reconstruction Methods

In MRI, the k-space is a mathematical representation of the raw data acquired during the imaging process. During an MRI scan, the imaging process involves the use of strong magnetic fields and radiofrequency pulses to excite and manipulate hydrogen nuclei in the body. The resulting signals, known as echoes, are detected by the MRI machine. The raw data collected during an MRI scan can be represented in two domains: the spatial domain (image space) and the frequency domain (k-space). The spatial domain corresponds to the actual image space, while the k-space represents the spatial frequency information. The reconstructed image is generated from the transformed k-space data. Different regions of the k-space contribute to different image features. The center of the k-space contains low-frequency information that corresponds to the overall structure of the image, while the outer regions contain high-frequency details. Understanding the k-space is crucial for optimizing MRI acquisition parameters, designing efficient pulse sequences, and developing advanced reconstruction techniques. It is also relevant in the context of techniques such as parallel imaging, compressed sensing, and machine learning-based reconstruction methods that leverage k-space data for accelerated imaging. In general, the Fourier space or k-space describes the data acquired from the MRI scanner, and is denoted by:

*I* = *IFFT*(*K*) (1)

where *I* is the reconstructed image, *IFFT* is the inverse fast Fourier transform, and *K* is the k-space data. In case of PI-MRI, the acquired data from multiple coils are combined to reconstruct the image [79]. Let $S_{c}(p,q)$ represent the sensitivity profile of the c-th coil and $K_{c}(u,v)$ represent the k-space data acquired by the c-th coil. The combined k-space data *K*(*u*,*v*) are obtained by weighting and summing the data from each coil:

(2) $$K\left(u,v\right)=\sum_{c}S_{c}\left(p,q\right)\cdot \;K_{c}(u,v)$$

The final reconstructed image $I_{rec}\left(p,q\right)$ is obtained by applying the *IFFT* to the combined k-space data:

(3) $$I_{rec}\left(p,q\right)=\iint K(u,v)\cdot \;e^{2\pi i\left(up+vq\right)}du\;dv$$

Acquiring data across the entire k-space in MRI can be time-consuming. The time required for data acquisition is influenced by factors such as the number of phase-encoding steps, the repetition time, and the field of view. To address this issue and reduce scan times, various acceleration techniques are employed, and undersampling is one of the commonly used approaches. Undersampling involves acquiring only a subset of the k-space data, allowing for faster image acquisition. This can be expressed as:

(4) I=IFFT (K·M)

In this case, *M* is an undersampled distribution that generates an undersampled k-space via element-wise multiplication with a fully sampled k-space. In PI-MRI, undersampled k-space data $K_{under}(u,v)$ are obtained via element-wise multiplication of the mask *M*(*u*,*v*) with the fully sampled k-space data:

(5) $$K_{under}\left(u,v\right)=K\left(u,v\right)\cdot M(u,v)$$

Then, several approaches are used for reconstructing images from this undersampled k-space. DL is one of the vital approaches for generating high-quality images from undersampled data. DL methods emphasize real-time MRI reconstruction and accelerated imaging techniques to reduce scan times and enhance patient comfort for both the PI- and CS-MRI. Advanced DL architectures, including CNNs, RNNs, attention mechanisms, and generative models (GANs and VAEs), are continuing to be explored for improved MRI reconstruction. A DL model is trained by both fully and partially sampled k-space data with corresponding images. Two training approaches are used in DL: supervised and unsupervised. Reinforcement learning (RL) [80] is also used in MRI processing. Image reconstruction methods using DL are classified as single- or multi-domain.

### 4.1. Single Domain Approach

**Figure 6:**
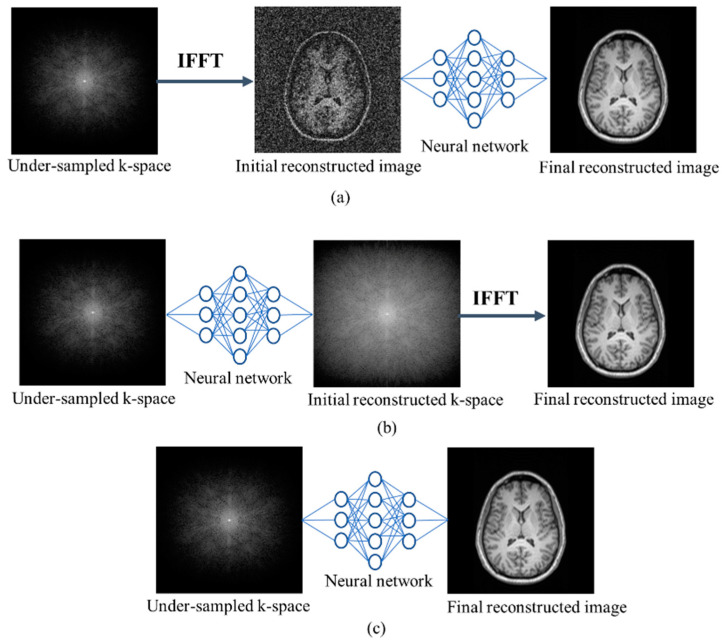
Single-domain MRI reconstruction methods: (**a**) image domain, (**b**) k-space domain, and (**c**) direct mapping. *IFFT*, inverse fast Fourier transformation.

A single-domain method is a reconstruction architecture that uses a single NN; an image space or k-space domain. Figure 6a–c show the functionality of an NN of the three-single-domains methodology. The usability of the image domain (Figure 6a) is similar to that of DL-based conventional (non-medical) image processing. Several image enhancement operations—such as denoising, super-resolution, and de-aliasing—can be performed using prior knowledge from large training datasets. The image is first reconstructed from zero-filled k-space data via *IFFT* and then a DL approach is applied to this reconstructed image. A deep cascade CNN architecture [81] independently reconstructs dynamic sequences of two-dimensional (2D) myocardial MRIs from every frame. Yang et al. [82] combined adversarial and innovative content losses but calculated the FFT of magnitude images instead of MRI raw data. Quan et al. [83] measured the cyclic loss using an autoencoder and GAN-based fully residual network, but only with training datasets. To discriminate between channels and lessen the background noise, Li et al. [84] presented a channel attention mechanism that combines dilated residual networks with a GAN. Phase-contrast MRI reconstruction [85] encodes low-frequency sections in the phase direction, although high frequencies are essential for storing image edges. K-space sampling strategies [86] play a crucial role in MRI and directly impact the quality and efficiency of image reconstruction. The k-space represents the spatial frequency information of the imaged object and is sampled during the MRI data acquisition process. Cartesian and non-Cartesian (random, Poisson-disc) are some common k-space sampling strategies [87]. A new sample pattern that combines the random and non-random frequencies of the phase direction was proposed by Hossain et al. [88]. Additionally, they developed an enhanced fully dense NN, which employs attention gates to eliminate redundant features. A U-net-based fused attentive-GAN [89] and super-resolution-GAN [90] have been applied to local fusion feature blocks to increase the image resolution. These methods enable only qualitative measurement of reconstructed images. Pixel-wise maps of reconstructed single-coil knee images based on VAE were reported [91], although perceptual mapping is important for the contextual and edge details of an image. To restore the fine details and eliminate noise, both global and local viewpoints are proposed by Gao et al. [92] but have high computing requirements and limited generalization to unknown data and imaging settings. However, the increased resolution hampers a full diagnosis of parts, which is essential for radiology.

The DL model in the sensor domain (Figure 6b) is used to estimate the abandoned frequencies. Multiple-slice k-space learning [93] interpolates a k-space based on different adjacent slices but does not recombine the features of these slices. An adaptive CNN [94] applies a residual encoder–decoder network using complementary information of spatially adjacent slices. However, this method is sensitive to changes in noise levels and acquisition specifications. K-space learning [95] uses a fully data-driven technique for k-space interpolation based on the low-rank Hankel matrix method [96] that interpolates the adjacent slices independently. The active MRI k-space [97] is trained using a fixed number of low frequencies but overlooks the issues of MRI phase-encoding sampling. RAKI [98] is a scan-specific approach that trains both linear and non-linear components based on the ResNet architecture, but it uses fixed learning rates that are not ideal for all situations. LORAKI [99] used RNNs to restore lost k-space data based on a scan-specific approach that trains the autocalibration signal by updating the weight for each k-space input data; as a result, this method requires more time for computation. Using a recurrent variational network, high-fidelity multi-coil MRI restoration is proposed [100], but it needs more memory during training to gather gradients for back-propagation for computing the loss function.

The transformation of under-sampled k-space data into uncorrupted images can be learned directly (Figure 6c). Zhu et al. [101] performed training using a large database of paired synthesized undersampled input data via a feed-forward DNN with fully connected layers and reconstructed the desired output images. Due to the large memory requirements of the fully connected layers, this method recovered images without interpolating the missing k-space samples. As a result, the images reconstructed using this method have noise and artifacts. Additionally, it is suitable only for relatively small images. By addressing this issue, an end-to-end MRI reconstruction (ETER-net) [102] proposed an architecture based on the RNN. Compared to fully connected architectures, this proposed scheme reconstructs images from k-space data with fewer parameters using the Cartesian trajectory. However, the RNN used in the ETER-net included characteristics only in the horizontal and vertical dimensions, which could affect performance.

### 4.2. Multi Domain Approach

**Figure 7:**
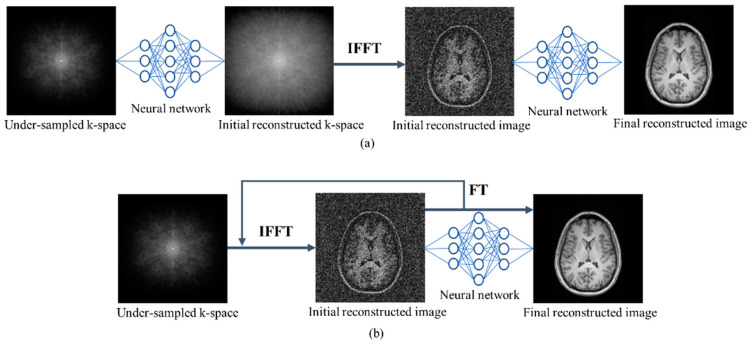
Multi-domain MRI reconstruction methods: (**a**) multi-domain and (**b**) iterative optimization. FT, Fourier transform.

A cross-domain (Figure 7a) method operates in both the frequency and image domains. The frequency/sensor domain network attempts to estimate the unacquired frequencies; subsequently, the network of the spatial domain performs the image enhancement operation. KIKI-net [103] and hybrid-cascade-net [104] encapsulate data-consistency layers to train both domains. Hybrid-cascade-net applied six CNN blocks: two for the sensor domain and four for the next domain. By contrast, KIKI-net used four CNNs, and each network contained 100 convolutional layers and was trained independently to generate random sampling points in the k-space. However, they used magnitude images from an undersampled k-space as input instead of raw k-space data, which could have an impact on performance. Dynamic cardiac MRI sequences were reconstructed by combining the temporal sequence dependencies [105]. The dual-domain cascade [106] reconstructs one image per channel via the sum-of-squares method using four Unets in each channel. The correlation between the image and frequency domains with variable consistency is described by a dual-domain deep lattice network [107]. IKWI-net [108] accepts both zero-filled k-space and images as input by applying four CNNs in the image, k-space, wavelet, and image domains. Multi-domain-CNN [109] used ResNet for k-space interpolation via multiple convolutional kernels and then a Unet was applied to reconstruct radial cardiac MRI. Ran et al. [110] developed the MRI dual-domain network (MD-Recon-Net) to investigate the implicit connection between spatial data and the k-space, but it restricted extrapolation to unknown data and imaging circumstances. The double-domain GAN [111] method preserves structural features and eliminate aliasing artifacts, but it is limited to clinical usability validation. Although these multi-domain models reconstructed high-quality images until now, they require a long time to train raw and image data. Figure 7 shows the operation of an NN as a component of a multi-domain methodology.

The iterative unrolled optimization method (Figure 7b) translates the measured k-space to the appropriate reconstructed image via unrolling iterations. The image transformation, sparsity-promoting functions, regularization parameters, and update rates can be viewed explicitly or implicitly, and back-propagation is used to fit them during training. Compared to conventional optimization, this method is more suited for learning image features. Model learning [112] gradually reduces the constraints using three convolutional layers in each primal–dual network block. A deep unrolling network [113] employs a variational architecture to capture image redundancy, which is built up of interleaved CNN blocks. A dense RNN [114] uses a multi-coil fastMRI knee dataset by applying a smaller number of iterations than the proximal gradient descent. However, over-imposing sparsity or penalties can produce cartoons or staircase artifacts. Jain et al. [115] developed an ideal representation of the magnitude and phase information in the data by using complex-valued operations on an iterative optimization network for MRI reconstruction, but it restricted extrapolation to unknown data and imaging circumstances. Non-trivial normalization methods and hyper-parameters must be selected carefully for optimization-based techniques. Due to the iteration, the reconstruction rates of these methods are typically slow.

### 4.3. Transformer-Based Reconstruction

**Figure 8:**
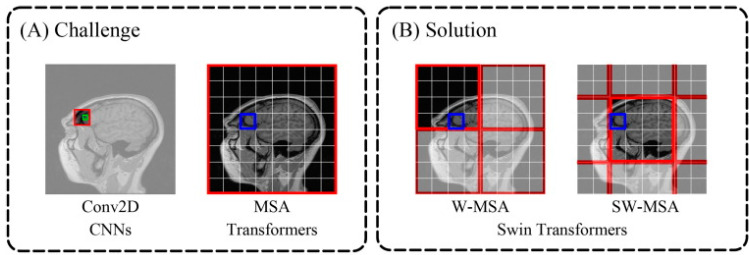
Schematics of the receptive fields for Conv2D, MSA, and W-MSA/SW-MSA are shown in (**A**,**B**). Red box, receptive field of the operation; green box, pixel; blue box, patch in self-attention.

Because convolutions are efficient feature extractors, CNNs have long held a privileged place in CV. The GAN-based model and many DL-based MRI reconstruction techniques are based on CNNs. Convolution, which is locally sensitive and independent of distance, provides the foundation for CNN feature extraction. CNNs’ receptive fields are constrained by the network depth and convolutional kernel. A large convolutional kernel increases the computing costs significantly, and a deep network can result in gradient vanishing. A transformer [116] is an NN architecture introduced in 2017. It was originally designed for machine translation but has since been applied to a wide range of NLP tasks, such as language modeling, summarization, and question-answering. Numerous studies have used a vision transformer or its modifications for MRI reconstruction after it demonstrated good performance in the CV sector. For instance, a Swin transformer reconstruction network was the foundation of a cascade framework created by Huang et al. [117] that considerably improved the image quality. The disadvantage of 2D convolution (Conv2D) and the concept of multi-head self-attention (MSA) are shown in Figure 8A [117]. The advantage of shifted windows-based multi-head self-attention (W-MSA/SW-MSA) is shown in Figure 8B. Conv2D lacks long-range dependency and is locally sensitive. The receptive fields of MSA and (S)WMSA are greater than those of Conv2D. W-MSA and SW-MSA are alternatively used in Swin transformers and executed in shifted windows; MSA operates in the entire image space.

Zhou et al. [118] suggested a combined image and k-space domain self-supervised learning method, which improved the reconstruction outcomes, to train a transformer in a self-supervised strategy. To accomplish the zero-shot reconstruction of undersampled data via optimizing the network parameters and latent and noisy variables, an unsupervised MRI reconstruction approach based on a zero-shot learning adversarial transformer was developed by Korkmaz et al. [119]. Liu et al. [120] used a deep data consistency block and a spatial attention selection module to restore MRI images with missing data recovered, but it lacked generalization and needed high processing costs. For the network to be as consistent as possible with undersampled MRI data, a transformer and a contrastive training strategy were merged. Huang et al. [121] created an edge-enhanced GAN-based Swin transformer network and a texture-enhanced GAN-based Swin transformer network to capitalize on the advantages of the transformer and GAN architectures for MRI reconstruction. Lyu et al. [122] used a multi-view GAN transformer to recreate the cardiac MRI, but it is not appropriate for PI and necessitates a lot of network settings. There are challenges when using transformer-based models for image reconstruction, such as handling high-resolution images and maintaining the spatial coherence of the output. Nonetheless, recent research has indicated that transformer-based models have potential for image reconstruction.

### 4.4. DL-Based 3D Reconstruction

DL-based three-dimensional (3D) reconstruction techniques [123,124] leverage NNs to infer the 3D structure of objects or scenes from 2D images or other input data. Recently, these techniques have gained popularity in CV and computer graphics due to their ability to generate detailed and accurate 3D shapes. DL-based techniques [125,126] are applied in the reconstruction of 3D MRI data to enhance the speed, quality, and efficiency of the imaging process. 3D CNNs [127,128] are used to directly learn the mapping from undersampled or corrupted MRI data to fully sampled or high-quality images. VAEs are trained to generate realistic 3D MRI volumes and subsequently used for reconstruction tasks [129]. GANs provide a way to enhance the resolution of 3D MRI volumes by leveraging advancements in 2D GAN super-resolution techniques [130]. The effect of this model is contingent on the ability of the 2D GAN to effectively learn and generate high-quality, realistic details in the MRI slices. The hybrid model [131] combining VAE and GAN tried to generate high-quality and realistic 3D MRI volumes while also ensuring that the generated volumes adhered to the distribution learned by the VAE. Recurrent GAN [132] is employed to capture temporal dependencies in dynamic MRI sequences, aiding in the reconstruction of moving structures. Attention mechanisms, such as self-attention or transformer-based architectures [133], are applied to capture long-range dependencies in 3D MRI data, improving the reconstruction quality. It is worth noting that the choice of the specific technique depends on the characteristics of the MRI data, such as whether the data are static or dynamic, fully sampled or undersampled, and the imaging modality (e.g., structural, functional, or diffusion MRI). Researchers continue to explore new architectures and methods to further advance the field of deep learning-based 3D MRI reconstruction.

## 5. Datasets and Source Codes

In this section, we describe the publicly available open-source codes of several DL-based MRI reconstruction methods and their datasets.

### 5.1. Datasets

When applying DL algorithms to a given area, data scarcity is a typical issue, and it is exacerbated in the case of medical image interpretation. Most researchers employing DL approaches to medical image analysis algorithms are computer scientists. Medical data are typically owned by institutions, which are unable to make them public due to privacy and ethics concerns. A major challenge in DL research is the large size of public datasets, especially those of MRI. Several protocols are used to store medical data. The brain imaging data structure (BIDS) and neuroimaging informatics technology initiative (NIFTI) are standards for MRI brain datasets. The digital imaging and communications in medicine (DICOM) protocol is commonly used to store, transmit, process, and display medical images. The International Society for Magnetic Resonance in Medicine (ISMRM) [134] provides an MRI raw data standard. The performance and accuracy of a DL-based algorithm depend on proper data collection and preprocessing. Several institutions and academics have organized DL-based medical image analysis competitions [135,136] to encourage computer-assisted medical image processing. Additionally, they have published medical imaging datasets for a variety of purposes. The medical image computing and computer-assisted intervention (MICCAI) [135] is an organization that aimed at identifying the underlying technologies in a wide range of applications, promoting their technical and clinical validation, and collaborating with physicians and medical associations to set evaluation criteria. FastMRI [136] provides fully sampled single- and multi-coil MRI raw knee and brain data, and DICOM images obtained using 1.5 and 3 Tesla (T) magnetic fields. OpenNeuro [137] contains MRI and electroencephalography (EEG) neuroimages based on the BIDS protocol. The Autism Brain Imaging Data Exchange (ABIDE) [138] contains functional MRI data. The Open Access Series of Imaging Studies (OASIS) [139] contains brain MRI data created at Washington University. The Human Connectome Project (HCP) [140] provides brain MRIs obtained using four imaging modalities with 3 and 7 T magnets. Calgary-Campinas [141] offers T1-weighted brain MRI datasets obtained using 1.5 and 3 T magnets. Brain tumor segmentation (Brats) [142] stores T1 and T2 brain MR images focused on glioma segmentation. Mridata [143] contains fully sampled complex-valued k-space raw knee data from several vendors. IXI [144] stores T1, T2, PD, and diffusion tensor imaging (DTI) brain MRIs in NIFTI format. The Internet Brain Segmentation Repository (IBSR) [145] contains T1-weighted MRI brain images. The main objective of the Brats, OASIS and IXI datasets is image segmentation, but they are also used for MRI reconstruction tasks. However, many of the DL-based MRI reconstruction methods simulate k-space data from the FFT of images instead of MRI raw data.

### 5.2. Open-Source Codes

Several DL-based open-source codes have been implemented to reconstruct MRI, most in Python programming languages. Table 2 lists the open-source codes of DNNs for MRI reconstruction together with the network type, DL tool, datasets, and input domain. Among them, some methods are used for motion artifact correction.

## 6. Implementation Challenges and Future Perspectives

Deep learning architectures have demonstrated remarkable capabilities in various clinical applications, including MRI image reconstruction. State-of-the-art DL models are often designed to address specific intents in clinical scenarios, providing improvements in accuracy, speed, and overall diagnostic capabilities. DNNs, VAEs, and GANs have accelerated the MRI acquisition times for clinical scans, reducing patient discomfort and improving the overall workflow efficiency. CNNs with attention mechanisms and U-net architectures have enhanced spatial resolution in MRI reconstructions, providing detailed images for the improved visualization of anatomical structures. Autoencoders and CNNs with residual connections have reduced the noise in MRI images, particularly in low signal-to-noise ratio (SNR) scenarios, to improve the image quality and diagnostic confidence. CNNs with attention mechanisms and GANs have mitigated the common artifacts in MRI, such as motion artifacts, aliasing artifacts, and susceptibility artifacts, to enhance diagnostic accuracy. The CycleGANs and U-net variants have generated MRI-like images from other imaging modalities (e.g., computed tomography) or synthesizing different MRI contrasts, aiding in multi-modal image analysis and clinical decision-making. RNNs and 3D CNNs have facilitated real-time reconstruction for dynamic imaging (e.g., cardiac imaging) and improved the temporal resolution of functional MRI (fMRI) studies. These clinical applications showcase the versatility of state-of-the-art DL architectures in addressing various tasks in relation to MRI reconstruction.

Despite technological breakthroughs in DL for MRI reconstruction, many challenges remain to be overcome [17]. Quantitative measurements such as the structural similarity index, mean-squared error, root-mean-squared error, and peak signal-to-noise ratio are frequently used to assess the performance of a network. However, in clinical applications, prospectively acquired data reconstructed using DL need to be evaluated for qualitative image quality, diagnostic scoring, and measurement of clinical metrics such as the image distortion, edge sharpness, and motion artifacts fidelity. In MRI, various artifacts can occur that may affect image quality and interpretation. Aliasing artifacts, motion artifacts, chemical shift artifacts, susceptibility artifacts, and radiofrequency artifacts are some common artifacts in MRI. These artifacts can arise from a range of sources, including patient-related factors, hardware issues, and imaging parameters. It is important to note that artifact mitigation strategies may vary depending on the specific MRI sequence and clinical scenario. DL models may have trouble with intricate artifacts that considerably deviate from the distribution of the training data. Model performance on artifacts can be enhanced by including a variety of artifacts in the training data, utilizing loss functions that are data-specific. Some studies employed only magnitude data, whereas others trained distinct networks for magnitude and phase data [172] or split the real and imaginary components into two channels [173]. These procedures do not always preserve the data’s phase information. The development of complex-valued networks [174] is a focus of research. It is difficult to compare methodologies and assess their robustness and generalizability because most studies report results obtained using their own datasets. The generalizability of DL models is constrained by their requirement for large, labeled training datasets, which can be difficult to acquire. Techniques for domain adaptation, transfer learning, and data augmentation can overcome the lack of data and enhance generalization. Because imaging features vary between scanners and methods, models that were trained on particular datasets may not perform well when applied to data from those other scanners or processes. Generalization across various imaging contexts can be improved through model ensemble approaches, federated learning, and domain-specific normalization techniques. DL simulations lack interpretability, transparency, and the capacity to offer thorough justifications for the outcomes of their estimations or reconstructions. Attention mechanisms, interpretability strategies, and incorporation with clinical information or rule-based algorithms are a few explainable AI techniques that can improve interpretability and produce explicable outputs. The lack of integration with the clinical environment is a barrier to successful DL reconstructions in MRI because of their computational complexity. A reduction in the number of computational resources needed can be achieved using model compression approaches, effective network topologies, and hardware acceleration. Concerns about the robustness and dependability of DL models are raised due to the possibility of adversarial attacks. Model robustness against adversarial attacks can be improved through adversarial training, input preprocessing (such as denoising and smoothing), and defensive methods (such as detection and certification). Hyperparameter settings affect DL model performance, necessitating careful tweaking. Effective hyperparameter tweaking can improve model performance.

We highlighted potential directions and trends of DL in MRI reconstruction based on current research and advancements. Figure 1 shows that this field is ever-evolving and that there have been fresh advancements implemented in the interim. As DL-based MRI reconstruction techniques grow and demonstrate their effectiveness in research settings, there is a likelihood of increased adoption in clinical practice. Advancements in computational power and algorithm efficiency may enable real-time MRI reconstruction for dynamic imaging applications, such as cardiac imaging and functional MRI (fMRI). This could significantly improve the ability to monitor physiological processes in real time. DL-based reconstruction may be integrated with other imaging modalities, such as positron emission tomography (PET) or computed tomography (CT), to provide comprehensive and fused imaging information. This integration could enhance diagnostic capabilities and improve patient care. Future models may focus on improving robustness to variations in imaging protocols and scanner types. This could facilitate the deployment of DL-based reconstruction techniques across diverse clinical environments with minimal tuning. DL models may continue to be developed to handle multi-contrast imaging scenarios efficiently. Diffusion-weighted MRI (DW-MRI) [175] is a specialized MRI technique that measures the diffusion of water molecules within tissues. The diffusion model [176] in this context helps to capture the spatial distribution and characteristics of water diffusion, which is particularly relevant in applications such as diffusion tensor imaging (DTI) and diffusion-weighted imaging. The ability to reconstruct different contrasts from the same acquired data could streamline imaging protocols and improve diagnostic information. Standardization efforts and collaborative initiatives across research institutions, industry, and regulatory bodies may emerge to establish guidelines and best practices for the development and deployment of DL-based MRI reconstruction.

## 7. Conclusions

This systematic review of deep learning-based compressed sensing MRI reconstruction reveals a growing body of literature exploring the synergies between deep learning methodologies and compressed sensing techniques. The integration of these two approaches shows promising results in addressing challenges associated with accelerated MRI scans, including reduced acquisition times and improved reconstruction quality. The reviewed studies demonstrate that deep learning models, such as CNNs and GANs, contribute to the efficient reconstruction of high-quality images from undersampled k-space data. The key findings indicate that DL-based methods outperform traditional CS approaches in terms of the reconstruction accuracy and robustness to undersampling artifacts. The ability of DL models to learn complex relationships within the data enables them to adapt to diverse imaging scenarios and improve the reconstruction quality across various anatomical structures.

Additionally, network training techniques such as TL and FL offer promising approaches for collaborative and data-efficient MRI reconstruction, respectively. These techniques help address challenges related to privacy, limited data, and generalization across diverse datasets in the medical imaging domain. Moreover, this review also highlights certain challenges and considerations. Issues such as the need for large and diverse datasets, fine-tuning for specific imaging protocols, and potential overfitting remain areas of concern. The promising outcomes of the reviewed studies suggest that deep learning-based compressed sensing MRI reconstruction has the potential to revolutionize the field by offering faster and more efficient imaging protocols. Future research can focus on addressing the current limitations, standardizing the evaluation metrics, and exploring the clinical translatability of these advanced reconstruction techniques.

## Figures and Tables

**Table 1:** Deep learning tools.

Ref.	Tool Name	Description
[43]	Deeplearning4j	Distributed deep learning library that allows for training models on Java interoperating with the Python environment.
[44]	Julia	A flexible and dynamic framework that is more suitable for scientific and numerical computing.
[45]	Keras	A Python-based library that is integrated with TensorFlow and used in different ML algorithms.
[46]	MatConvNet	A MATLAB toolbox used for image reconstruction, segmentation, and classification by CNN.
[47]	MS cognitive toolkit	Describes DNNs as a series of computationally directed graphs, where leaf nodes represent input parameters and other nodes indicate matrix operation.
[48]	Neural designer	Data mining tool that was developed by the Artelnics company used in NNs.
[49]	PyTorch	Developed by Facebook, works on complex data and is easy to learn.
[50]	Scikit-image	Applied for histogram equalization of the input images on various image processing algorithms.
[51]	Sigpy	The signal processing package operates on multi-dimensional array plotting and MRI reconstruction.
[52]	TensorFlow	Open-source Python framework developed by Google Brain Team that is the most used tool for developing deep learning models.
[53]	TensorFlow Federated (TFF)	An open-source framework developed by Google, TFF provides tools for FL. It allows developers to implement federated models and train them across distributed devices.
[54]	PySyft	PySyft is a flexible and powerful library for encrypted privacy-preserving ML. It extends PyTorch and TensorFlow to enable the security of FL.
[55]	Substra	In 2016, a multi-partner research project developed this FL framework. It concentrates on the medical industry to protect patient privacy and data ownership. It is currently utilized by the pharmaceutical industry for drug discovery.

**Table 2:** Open-source codes for MRI reconstruction.

Ref.	Network	DL Tool	Dataset	Domain
[146]	RL	PyTorch v0.3.1	fastMRI knee	Image
[147]	GAN	TensorFlow v1.4	Mridata	Image
[148]	RNN	PyTorch v0.4	Mridata	Dual/cross
[149]	RL	PyTorch v0.3	fastMRI	Sensor
[150]	CNN	MatConvNet v1.0-beta24	Mridata	Sensor
[151]	CNN	TensorFlow v1.11	Calgary-Campinas-359	Dual/cross
[152]	CNN	TensorFlow v1.7	Private knee and brain data	Iterative
[153]	CNN	Keras v2.0.4	OASIS brain data	Image
[154]	VAE	TensorFlow v1.15	Globus [155]	Iterative
[156]	CNN	TensorFlow v2.8	fastMRI, OASIS	Benchmarking
[157]	CNN	PyTorch v0.3	IBSR-18	Iterative
[158]	Densely attention CNN	TensorFlow v2.4	Brats, fastMRI, IXI	Image
[159]	Residual attention CNN	TensorFlow v2.4	Calgary-Campinas	Dual/cross
[160]	Swin Transformer	PyTorch v1.9	Calgary-Campinas, Brats	Iterative
[161]	TL, GAN	TensorFlow v2.3	Calgary-Campinas, Mridata	Image
[162]	FL	PyTorch v1.1	ABIDE	-
[163]	FL	PyTorch v1.7	fastMRI, Brats	-
[164]	Encoder-decoder	PyTorch v0.2	FastMRI knee	Image
[165]	GAN	TensorFlow v1.7	Brain data	Image
[166]	Encoder-decoder	TensorFlow, PyTorch	IXI, fastMRI	Benchmarking
[167]	VAE	TensorFlow v1.14	HCP	3D Imaging
[168]	VAE-GAN	PyTorch v0.4	Brats	3D Imaging
[169]	Unet	TensorFlow v2.0	Private MRI brain data	Motion artifact correction
[170]	Stacked Unet	TensorFlow v2.3		
[171]	CNN	MatConvNet v1.0-beta 19		

## Abbreviations

2D	Two-Dimensional
3D	Three-Dimensional
4D	Four-Dimensional
ABIDE	Autism Brain Imaging Data Exchange
BIDS	Brain Imaging Data Structure
Brats	Brain Tumor Segmentation
CNN	Convolutional Neural Network
CS	Compressed Sensing
CS-MRI	Compressed Sensing-Magnetic Resonance Imaging
DICOM	Digital Imaging and Communications in Medicine
DL	Deep Learning
DNN	Deep Neural Network
DTI	Diffusion Tensor Imaging
FDA-CNN	Fully Dense Attention CNN
FL	Federated Learning
GAN	Generative Adversarial Network
HCP	Human Connectome Project
IBSR	Internet Brain Segmentation Repository
IFFT	Inverse Fast Fourier Transform
ISMRM	International Society For Magnetic Resonance In Medicine
LSTM	Long Short-Term Memory
MICCAI	Medical Image Computing And Computer-Assisted Intervention
MRI	Magnetic Resonance Imaging
MSA	Multi-Head Self-Attention
NIFTI	Neuroimaging Informatics Technology Initiative
NLP	Natural Language Processing
NN	Neural Network
OASIS	Open Access Series of Imaging Studies
PI	Parallel Imaging
PRISMA	Preferred Reporting Items for Systematic Reviews and Meta-Analyses
RA-CNN	Residual Attention CNN
RL	Reinforcement Learning
RNN	Recurrent Neural Network
Swin	Shifted Windows
SW-MSA	Shifted Windows-based Multi-head Self-Attention
T	Tesla
TL	Transfer Learning
VAE	Variational Autoencoder
